# Whole exome sequencing as a diagnostic tool for patients with ciliopathy-like phenotypes

**DOI:** 10.1371/journal.pone.0183081

**Published:** 2017-08-11

**Authors:** Sheila Castro-Sánchez, María Álvarez-Satta, Mohamed A. Tohamy, Sergi Beltran, Sophia Derdak, Diana Valverde

**Affiliations:** 1 Departamento de Bioquímica, Genética e Inmunología, Facultad de Biología, Universidad de Vigo, Vigo, Spain; 2 Research Group of Rare Diseases & Pediatric Medicine, Instituto de Investigación Sanitaria Galicia Sur (IISGS), SERGAS-UVIGO, Hospital Álvaro Cunqueiro, Vigo, Spain; 3 Centro de Investigaciones Biomédicas (CINBIO) (Centro Singular de Investigación de Galicia), Universidad de Vigo, Vigo, Spain; 4 CNAG-CRG, Centre for Genomic Regulation (CRG), Barcelona Institute of Science and Technology (BIST), Barcelona, Spain; 5 Universitat Pompeu Fabra (UPF), Barcelona, Spain; National Eye Institute, UNITED STATES

## Abstract

Ciliopathies are a group of rare disorders characterized by a high genetic and phenotypic variability, which complicates their molecular diagnosis. Hence the need to use the latest powerful approaches to faster identify the genetic defect in these patients. We applied whole exome sequencing to six consanguineous families clinically diagnosed with ciliopathy-like disease, and for which mutations in predominant Bardet-Biedl syndrome (*BBS*) genes had previously been excluded. Our strategy, based on first applying several filters to ciliary variants and using many of the bioinformatics tools available, allowed us to identify causal mutations in *BBS2*, *ALMS1* and *CRB1* genes in four families, thus confirming the molecular diagnosis of ciliopathy. In the remaining two families, after first rejecting the presence of pathogenic variants in common cilia-related genes, we adopted a new filtering strategy combined with prioritisation tools to rank the final candidate genes for each case. Thus, we propose *CORO2B*, *LMO7* and *ZNF17* as novel candidate ciliary genes, but further functional studies will be needed to confirm their role. Our data show the usefulness of this strategy to diagnose patients with unclear phenotypes, and therefore the success of applying such technologies to achieve a rapid and reliable molecular diagnosis, improving genetic counselling for these patients. In addition, the described pipeline also highlights the common pitfalls associated to the large volume of data we have to face and the difficulty of assigning a functional role to these changes, hence the importance of designing the most appropriate strategy according to each case.

## Introduction

Over the last 15 years, our knowledge about the group of human genetic diseases called ciliopathies has rapidly grown [1, 2]. These multisystemic disorders, characterized by ciliary dysfunction, are caused by mutations in highly conserved genes mainly involved in the correct assembly and maintenance of cilia [3, 4].

It is well-known that one ciliary gene can be involved in the development of two or more distinct disorders, such as *MKKS*, which may be related to McKusick-Kauffman syndrome (MKKS, MIM #236700) or Bardet-Biedl syndrome (BBS, MIM #209900). This genetic heterogeneity, together with the high phenotypic variability, overlapping phenotypes and the progressive onset of many features during childhood and adolescence, complicates both the clinical and molecular diagnosis [1, 5].

In this respect, genetic tools have evolved considerably over the years. These syndromes were initially confirmed by the time-consuming Sanger sequencing, often combined with linkage analysis and autozygosity mapping strategies, or by DNA microarrays, which are restricted to a previously defined set of polymorphisms and rarely allow for the detection of rare/novel mutations. Thus, the shortcomings associated with these methods have encouraged most researchers to take advantage of next generation sequencing technologies [6, 7]. These approaches have emerged as a powerful way to improve molecular diagnosis in ciliopathy-related families.

Today, the ever lower cost of these techniques has promoted the implementation of these tools in molecular diagnosis laboratories and has led to an increased identification of novel ciliary-related genes in the last few years [8, 9]. It is worthwhile using WES as diagnostic tool since it allows us to get the mutational screening of nearly all coding regions of the genome at once, especially in such heterogeneous diseases as most of ciliopathies [4, 7, 10, 11].

However, the main challenge is how to interpret the great volume of resulting data, since variants with clinical relevance are difficult to pinpoint considering the high number of polymorphisms and possible false positives [5, 10]. This arduous task is being addressed thanks to the range of bioinformatics tools which help to adopt different strategies to narrow down the list of candidate variants [12, 13], rejecting and prioritizing variations by applying different filters based on (i) mode of inheritance, (ii) absence in public databases, (iii) predicted pathogenicity, (iv) role of the affected gene in pathways of interest or (v) impact on protein-protein interactions, among others.

The described identification of genes strongly related to cilia and the need to obtain an accurate diagnosis prompted us to perform whole exome sequencing (WES) in six consanguineous families with a clinical diagnosis of ciliopathy-like disorders, primarily BBS. The main objective of this study is to analyse massive sequencing data from these affected families following a strategy based on different filters like those mentioned above, as well as the use of some prioritisation tools (as described in “Materials and methods” section) on a reduced list of candidate genes, enhancing the possibilities to identify the causative mutation in each case.

## Materials and methods

### Patients

Six patients from unrelated families with clinical diagnosis of cilia-related disease were included. Consanguineous relationships have been described for all these families, which are from Western Europe, Africa and Asia. This group of patients consisted of five women and one man. When pedigree information was acquired, peripheral blood from all participants and available family members was collected for DNA extraction using the Flexigene DNA kit 250 (Qiagen, Hilden, Germany), following the manufacturer’s protocol. After a prior analysis by direct sequencing to exclude mutations in predominant *BBS* genes, *BBS1* and *BBS10*, and also *BBS12* according to our cohort data [14], the selected patients were studied by WES. In case 5, *MKKS/BBS6* gene was also previously sequenced considering the phenotype displayed. Genomic DNA fulfilled the quality criteria required for WES.

This study was approved by the Galician Ethical Committee for Clinical Research (Spain—no.2006/08) and adhered to the tenets of the Declaration of Helsinki. Written informed consent was obtained from all patients or their guardians.

### Library preparation and sequencing

Exome sequencing and analysis was performed at the Centro Nacional de Análisis Genómico (CNAG-CRG, Barcelona, Spain). For exome enrichment the NimbleGen SeqCap EZ v3.0 system following manufacturer's protocol version 4.2 was used and pre-capture multiplexing was applied. Briefly, 1 μg of genomic DNA was fragmented with Covaris ^™^E210 and used for ligation of the adapters containing Illumina specific indexes with a KAPA Library Preparation kit (Kapa Biosystems). Adapter ligated DNA fragments were enriched by 7 cycles of pre-capture PCR using KAPA HiFi HotStart ReadyMix (2X) (Kapa Biosystems) and analysed on an Agilent 2100 Bioanalyzer with the DNA 1000 assay. Five libraries were pooled with a combined mass of 1250 ng for the baits hybridisation step (47°C; 68 h). After washing (47°C), multiplexed captured library was recovered with Capture beads and amplified with 14 cycles of post-capture PCR using KAPA HiFi HotStart ReadyMix (2X). Size, concentration and quality of the captured library were determined using an Agilent DNA 1000 chip. The success of the enrichment was measured by qPCR SYBR Green assay on a Roche LightCycler^®^ 480 Instrument evaluating one genomic locus with pre- and post-captured material.

Each library pool was sequenced on an Illumina HiSeq 2000 instrument in a fraction of a sequencing lane following the manufacturer’s protocol, with a paired end run of 2x101bp. Image analysis, base calling and quality scoring of the run were processed using the manufacturer’s software Real Time Analysis (RTA 1.13.48) and followed by generation of FASTQ sequence files by CASAVA.

### Data analysis

Sequencing reads were trimmed from the 3’ end up to the first base with a Phred quality >9 and were mapped to the Human Genome Reference v37 with decoy sequences (Broad Institute), using GEM [15]. BAM files containing only properly paired and uniquely mapped reads were processed with picard tools v1.110 to remove duplicates, and local realignment was performed with the Genome Analysis Tool Kit (GATK) v3.1 [16]. Samtools v0.1.19 [17] was used on the processed BAM files to call single nucleotide variants (SNVs) and small insertion deletions (INDELs). Functional annotations from Ensembl release 75 [18] were added to the resulting Variant Call Format (VCF) file using snpEff [19]. snpSift [20] was used to add information from dbSNP v137 [21], the 1000 Genomes Project (1000GP) [22], the NHLBI Exome Sequencing Project [Exome Variant Server, NHLBI GO Exome Sequencing Project (ESP), Seattle, WA] and a variety of conservation and deleteriousness predictions included in dbNSFP v2.5 [23].

### Prioritisation of genetic variants

**Fig 1:**
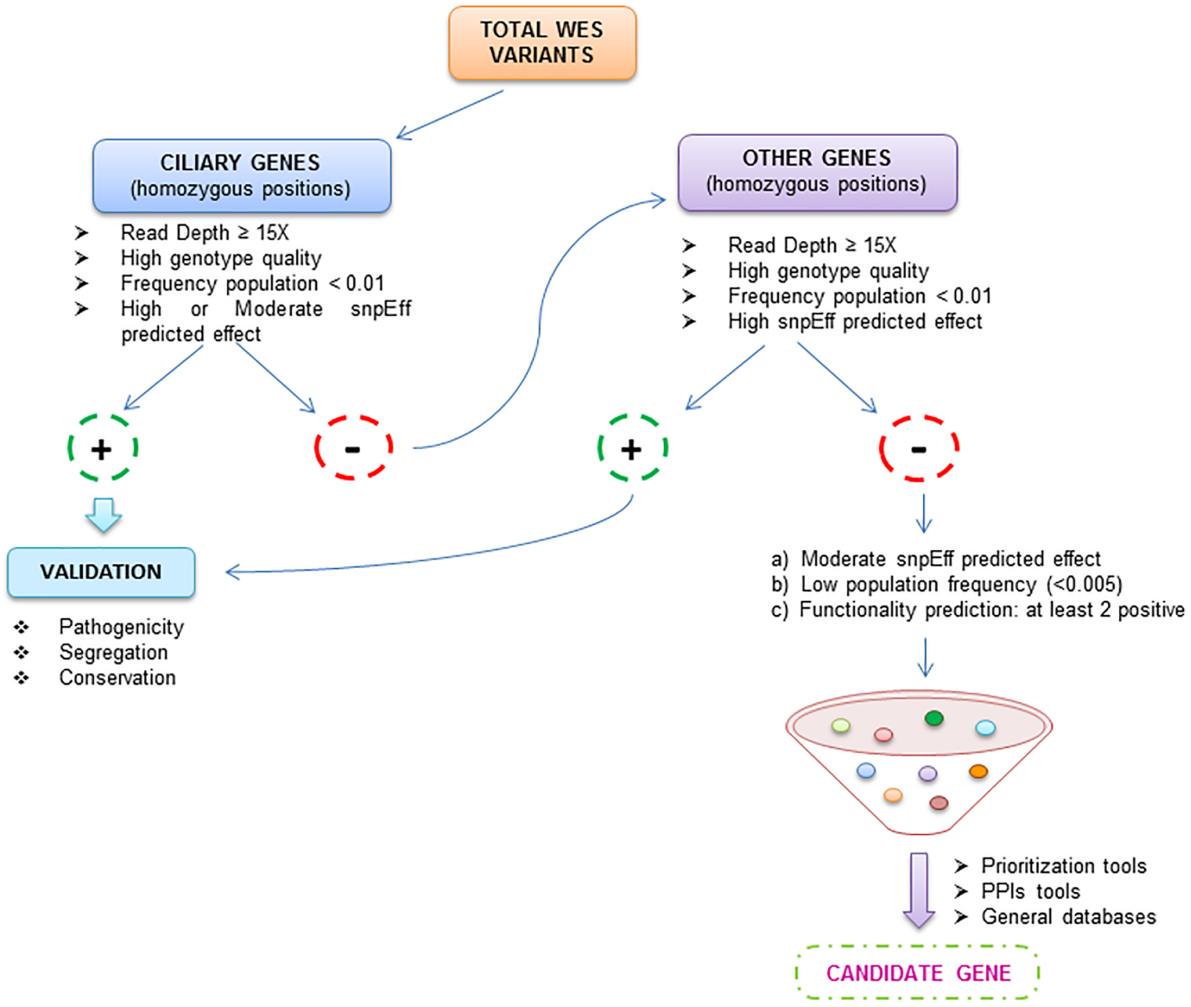
Workflow of the study.

This process was carried out following a strategy of our own design to reach a small number of candidate variants (Fig 1). Homozygous variants in known cilia-related genes were analysed first, as well as compound heterozygous variants in these genes, and then, we proceeded in the same way with homozygous variants in other genes. Only positions with a coverage of at least 15X and a genotype quality >20 (indicating only confident positions) were considered. Subsequently, variants with a minor allele frequency >0.01 in the dbSNP, and also with an alternative allele frequency >0.01 in NHLBI ESP or 1000GP databases were excluded. Next, based on functional annotation, variants with a snpEff predicted high or moderate effect were kept, whereas synonymous coding and intronic variants not associated with splice site alterations were excluded. Finally, the variants selected in the previous step were evaluated with several *in silico* prediction algorithms to evaluate the predicted effect at protein level (PolyPhen-2 [24], SIFT [25], Mutation Taster [26] and the likelihood ratio test (LRT) [27]). Once potentially pathogenic variants were selected, COBALT [28] was used to analyse protein residue conservation across species. Additionally, Endeavour [29] and ToppGene Suite [30] prioritisation tools were used to rank the final list of variants, with a training set of 51 known ciliary genes (S1 Table). For those top genes, STRING [31] and GENEMANIA [32] tools were used to elucidate possible co-expression and/or interactions between the corresponding encoded proteins and other ciliary proteins. When appropriate, the potential effect on splice sites was assessed using several prediction tools: NNSplice [33], NetGene2 [34], Human Splicing Finder [35] and ASSEDA [36], all with default settings. Finally, WES data were used to check if candidate variants localize in runs of homozygosity (ROH) regions, what is expected in consanguineous cases. Thus, homozygosity mapping with exome data was carried out with PLINK v1.9 [37] using optimized settings for Exome data [38].

### Confirmation of variants and segregation analysis

Selected candidate causative variants were verified by PCR amplification of the corresponding coding and adjacent intronic regions following standard protocols. Purified DNA fragments were directly sequenced using BigDye Terminator v.3.1 Cycle Sequencing Kit (Life Technologies, Foster City, CA, USA) and analysed on an ABI PRISM 3130 genetic analyser (Life Technologies, Foster City, CA, USA). Validated mutations were then analysed in the available family members to confirm co-segregation.

## Results

### Clinical findings

All the cases included in this study were clinically diagnosed with some type of ciliopathy, mainly BBS. Case 1 is a Caucasian female fulfilling diagnostic criteria for BBS, showing five out of the six primary features established for this disease: retinal dystrophy, obesity, polydactyly, urogenital anomalies and cognitive impairment [39]. Case 2, a female of Algerian origin, also displayed the same BBS primary features as case 1, in addition to several secondary features such as brachydactyly in four limbs, syndactyly in feet, craniofacial anomalies, psychomotor and speech delay, late menarche, hearing loss and hypothyroidism. Case 3 is an Indian male with clinically diagnosed BBS, showing mild retinal dystrophy, obesity, postaxial polydactyly in four limbs, pulmonary artery stenosis and some craniofacial defects. Case 4, an Iranian female, displayed retinal dystrophy, mild obesity, cognitive impairment and several facial anomalies (deep-set eyes, orbital hypertelorism and downward slanting palpebral fissures, among others), who did not strictly fulfilled diagnostic criteria but seemed to be closely related to BBS. Case 5, a Moroccan female child, was diagnosed with MKKS at birth since she showed postaxial polydactyly and hydrometrocolpos, together with vaginal agenesis. Case 6 belongs to a Caucasian family with an initial clinical diagnosis of BBS and consists of two affected siblings. At the time of submitting all samples to WES, only the sample of one of the siblings was available, a female who displayed retinal dystrophy and obesity. While WES data was being analysed, the phenotype reassessment of this family by clinicians only confirmed retinal dystrophy in the studied patient, whereas her brother was seen to fulfil diagnostic criteria of a more complex ciliopathy like BBS, showing retinal dystrophy, obesity, polydactyly and cognitive impairment. The main clinical data are summarized in Table 1.

**Table 1:** Summary of clinical data and molecular findings of the patients included in this study.

Patient ID	Gender	Ethnicity	Age (yo)	RD	OB	LA	UA	RA	CI	CA	Other	Gene	Variant^^^	Reference	Segregation^Δ^
**Case 1**	F	Caucasian (Germany)	31	+	+	+	+	-	+	-		*CORO2B*	c.581T>A; p.(Leu194Gln)	ExAC database^¥^	Yes (M)
												*SLC3A1*	c.1381T>C; p.(Tyr461His)	Endsley et al., 1997 [40]	
**Case 2**	F	African (Algeria)	14	+	+	+	+	-	+	+	PSD, LM, SHL, HT	*BBS2*	c.565C>T; p.(Arg189*)	Smaoui et al., 2006 [41]	Yes (M)
**Case 3**	M	Asian (India)	6	+ (mild)	+	+	-	NA	-	+	PAS	*BBS2*	c.1932T>A; p.(Tyr644*)	This study	Yes (M, F)
**Case 4**	F	Middle Eastern (Iran)	NA	+	+ (mild)	-	NA	-	+	+		*ALMS1*	c.8005C>T; p.(Arg2669*)	Bond et al., 2005 [42]	NA
**Case 5**	F	African (Morocco)	NA	-	-	+	+	-	NA	-		*LMO7*	c.890C>T; p.(Pro297Leu)	ExAC database^¥^ (both)	NA
												*ZNF17*	c.1903G>T; p.(Glu635*)		
**Case 6**	F	Caucasian (Spain)	25	+	-	-	-	-	-	-		*CRB1*	c.613_619delATAGGAA; p.(Ile205Aspfs*13)	ExAC database^¥^	Yes (M, F, S)

Abbreviations: yo, years old; RD, retinal dystrophy; OB, obesity; LA, limb abnormalities; UA, urogenital anomalies; RA, renal abnormalities; CI, cognitive impairment; CA, craniofacial anomalies; PSD, psychomotor and speech delay; LM, late menarche; SHL, sensorial hearing loss; HT, hypothyroidism; PAS, pulmonary artery stenosis; NA, not available; M, mother; F, father; S, sibling.

^(+)^ means presence and (^-^) means absence.

^(^)^ All variants were found in homozygous state.

^(¥)^ ExAC database: Exome Aggregation Consortium (http://exac.broadinstitute.org/).

^(Δ)^ Segregation analysis confirmed the heterozygous state of the identified variants in all available family members, showed in brackets.

Reference sequences (GeneBank): *CORO2B*, NM_006091.4; *SLC3A1*, NM_000341.3; *BBS2*, NM_031885.3; *ALMS1*, NM_015120.4; *LMO7*, NM_015842.2; *ZNF17*, NM_006959.2; *CRB1*, NM_201253.2.

### Exome sequencing reveals causative mutations in cilia-related genes

Given the consanguineous nature of our families, we expected a homozygous mutation to be the cause of the disease in all cases, but the responsible gene not to be the same given the different phenotypes.

The filtering strategy based on first considering only homozygous variants in the most common ciliopathy genes, then selecting those with good sequencing quality and high effect on coding sequence, and excluding those with a high frequency in public databases, allowed us to identify three candidate variants in three different cases. Two homozygous nonsense variants were found in *BBS2* gene (RefSeq NM_031885.3): c.565C>T; p.(Arg189*) (exon 5) and c.1932T>A; p.(Tyr644*) (exon 16), a novel mutation, in cases 2 and 3, respectively. Another homozygous nonsense variant, c.8005C>T; p.(Arg2669*) (exon 10), was detected in *ALMS1* gene (RefSeq NM_015120.4) in case 4. Although we assumed homozygous nonsense variants to be pathogenic, we checked MutationTaster and LRT, obtaining a deleterious prediction for all three variants (Table 2). The two *BBS2* variants are located in residues which are highly conserved across species. *BBS2* encodes a 722-amino acid protein; therefore both nonsense *BBS2* variants would lead to a shorter protein whose function could presumably be altered or even be subject to nonsense-mediated decay (NMD). The same occurs with the *ALMS1* variant, in which the length of the normal 4169-amino acid protein would be reduced by almost half. After that, these variants were validated by Sanger sequencing and then, the segregation analysis, which was performed in available members of cases 2 and 3 (no family members available for case 4), confirmed the recessive inheritance of the mutations identified in both cases (Table 1). Anyway, compound heterozygosity in known ciliary genes was also checked, without finding convincing results.

**Table 2:** Pathogenicity assessment of the variants identified by WES.

**PREDICTION AT PROTEIN LEVEL**						
**Gene**	**Variant**	**PolyPhen-2**	**SIFT**	**Mutation Taster**	**LRT**	**SCORE**
***BBS2***	p.(Arg189*)	-	-	Disease-causing	Deleterious	2/4
	p.(Tyr644*)	-	-	Disease-causing	Deleterious	2/4
***ALMS1***	p.(Arg2669*)	-	-	Disease-causing	Deleterious	2/4
***CORO2B***	p.(Leu194Gln)	Probably damaging	Tolerated	Disease-causing	Deleterious	3/4
***SLC3A1***	p.(Tyr461His)	Probably damaging	Tolerated	Disease-causing	Deleterious	3/4
***LMO7***	p.(Pro297Leu)	Probably damaging	Damaging	Disease-causing	Deleterious	4/4
***ZNF17***	p.(Glu635*)	-	-	Disease-causing	Deleterious	2/4
**SPLICING ANALYSIS**						
**Gene**	**Variant**	**NNSplice**	**NetGene2**	**HSF**	**ASSEDA**	**SCORE**
***CRB1***	p.(Ile205Aspfs*13)	-	+	+	+	3/4

^+^, means a positive prediction (changes in donor/acceptor splice sites);

^-^, means: at the top of the table, that these tools cannot be used for nonsense variants, and at the bottom of the table, a negative predicted effect on splicing process. Score values indicate how many tools of the total showed a positive prediction. Reference sequences (GeneBank): *CORO2B*, NM_006091.4; *SLC3A1*, NM_000341.3; *BBS2*, NM_031885.3; *ALMS1*, NM_015120.4; *LMO7*, NM_015842.2; *ZNF17*, NM_006959.2; *CRB1*, NM_201253.2.

### Identification of novel candidate genes

In the remaining three families, no candidate causal variants were detected in the most commonly mutated cilia-related genes. Then, we extended the mutational search to all WES data, focusing again on a selection of homozygous variants fulfilling the same criteria proposed for the previous families as a starting point. On the resulting file containing a set of positions (~300) for each case, a series of exclusion steps were followed to obtain the final candidate variants.

#### Case 1

In this case, no candidate variants with a snpEff predicted high effect were found after following the general filtering strategy, so different filters were applied: (a) only variants with a predicted moderate effect associated (modifier or low effects excluded), (b) very low population frequency (<0.005) in public databases, and (c) at least two (out of four) positive predictions with functionality prediction tools. Only seven variants in seven different genes fulfilled all of these criteria; the genes were ranked with Endeavour and Toppgene based on functional similarity to a training gene list including those human ciliary genes reported in the SYSCILIA gold standard v1 [43]. *CORO2B* (coronin 2B) was the top candidate in both algorithms, followed by *SLC3A1* (solute carrier family 3 member 1) gene. To improve the robustness of these results, we also tried with subsets of training genes associated with BBS and MKS separately. For the first case, *SLC3A1* was the top gene followed by *CORO2B*, whereas for the second case the opposite occurred. Finally, we decided to train Toppgene with a set of 51 common ciliary genes, also included in SYSCILIA (S1 Table). *SLC3A1* was again the top gene in this setting, with *CORO2B* in the second position. Cildb database [44] was also consulted to gain more information about these genes not previously related to any ciliopathy. While coronin-2B protein has been identified in ciliary studies in *C*. *elegans*, *D*. *melanogaster* and *M*. *musculus*, the neutral and basic amino acid transport protein rBAT (SLC3A1) has no record related to the cilium to date. We also checked the existence of protein-protein interactions (PPIs) which could help to find relations between the proteins encoded by these candidate genes and other ciliary proteins. STRING and GENEMANIA tools revealed the co-expression of coronin-2B protein with BBS7 (Bardet-Biedl syndrome 7 protein) and FRZB/SFRP3 (secreted frizzled-related protein 3; belongs to the soluble frizzled-related proteins, sFRPS, that function as modulators of Wnt signalling) [31, 32]. FRZB/SFRP3 is co-expressed with BBS10 (Bardet-Biedl syndrome 10 protein) [45]. Furthermore, coronin-2B is known to interact with ACTA1 [46], an actin-binding protein, and also with coronin-2A [47], which in turn directly interacts with another actin-binding protein, ACTB [48]. SCL3A1 seems to have a direct interaction with another solute carrier member SLC4A1 (Band 3 anion transport protein), a Cl^-^/HCO_3_^-^ exchanger expressed in sensory cilia of olfactory epithelium [49].

The two homozygous variants identified, c.581T>A, p.(Leu194Gln), in *CORO2B* (RefSeq NM_006091.4) and c.1381T>C, p.(Tyr461His), in *SLC3A1* (RefSeq NM_000341.3), were confirmed by Sanger sequencing, as well as the autosomal recessive pattern of inheritance after segregation analysis in the only member available (mother) (Table 1). The second copy of these variants is assumed to be inherited via the proband’s father. Both variants were predicted to be pathogenic by three out of four bioinformatics prediction tools (Table 2).

Although none of these two genes has been previously related to any ciliopathy, these evidences suggest that *CORO2B* could be the causative gene in this family. The variant identified in *SLC3A1* has already been described in patients with cystinuria (#220100) [50, 51], but according to their phenotypes this variant cannot explain case 1 phenotype, that is why *SLC3A1* was excluded as causative gene.

#### Case 5

The same filters as in the previous case were applied, considering variants with both high and moderate snpEff predicted effect, narrowing the list down to 8 candidate genes. These were also prioritised by using different subsets of training genes, resulting in *LMO7* (LIM domain 7) and *ZNF17* (zinc finger protein 17) as top candidates. Cildb database revealed the detection of *ZNF17* in previous ciliary studies in *C*. *elegans*, but no evidence was found for *LMO7*. Tools predicting PPIs revealed a link between ZNF17 and TTC8/BBS8 (Tetratricopeptide repeat protein 8) via OFIP/KIAA0753 (OFD1 and FOR20 interacting protein), and also the involvement of LMO7 in the same pathway as ACTN2 (Alpha-actinin-2), an actin-binding protein which physically interacts with TTC8. All these proteins have been associated to either ciliary disease or pathways [52–54].

Both homozygous variants, c.890C>T; p.(Pro297Leu), in *LMO7* (RefSeq NM_015842.2) and c.1903G>T, p.(Glu635*); in *ZNF17* (RefSeq NM_006959.2), were confirmed by direct sequencing, but no family members were available to perform segregation analysis. All prediction tools classified these two variants as deleterious (Table 2).

#### Case 6

In this case, a homozygous deletion of 7 nucleotides, c.613_619delATAGGAA; p.(Ile205Aspfs*13), in *CRB1* (RefSeq NM_201253.2), a less common ciliary gene, was identified after examining those variants with a high impact prediction. *CRB1* has been previously related to retinitis pigmentosa and Leber Congenital Amaurosis [55, 56]. This variant has been assumed as deleterious since the protein presumably would be truncated, and also affects a highly conserved residue. Furthermore, since this change is located at the end of exon 2, and considering that *CRB1* gene contains 12 exons, we also analysed the putative effect on splice donor/acceptor sequences. Three out of four bioinformatics tools did predict an alteration of splicing efficiency, which reinforces the presumable pathogenicity of the variant (Table 2). Direct sequencing confirmed its presence in homozygous state in the studied patient (female). Segregation analysis in the parents supported the autosomal recessive inheritance of this variant (Table 1), and revealed its presence in heterozygous state in the patient’s sibling (male). This made us think in a more complex inheritance in the heterozygous carrier, but the negative results obtained in our previous genetic screening suggest that there should be an additional gene harbouring two mutated alleles, which could explain the phenotype displayed.

### ROH analysis from WES data

The analysis of WES data also identified ROHs consistent with the presence of consanguinity (ROH >100,000 kb in length) in all patients. In addition, we confirmed that all candidate variants localize within these genomic ROH regions. These results can be found in S2 Table.

## Discussion

Molecular diagnosis of rare diseases has evolved considerably over the last years, primarily due to the advent and subsequent improvements in next-generation sequencing technologies. Until recently, traditional Sanger sequencing was the most recurrent approach, involving long working hours, particularly when analysing large genes or multigenic diseases. This, together with the high genetic heterogeneity associated with these disorders and the frequent difficulty to obtain an accurate clinical assessment, has hampered the molecular diagnosis, and therefore the possibility for patients to receive an appropriate genetic counselling and treatment [8, 55, 57].

Here we report the molecular analysis using whole-exome sequencing of six consanguineous families with different genetic background and primary clinical diagnosis of a ciliopathy, mainly BBS. The initial revision of clinical features showed a variety of phenotypes, among which only three patients fulfilled the BBS diagnostic criteria [39]. Therefore, we followed the molecular algorithm previously proposed by our group [58] consisting in first testing for prevalent mutations in BBS, whether through the BBS/AS Asper Ophthalmics genotyping microarray (Asper Biotech; Tartu, Estonia) or direct sequencing of the main genes involved. In the particular case of BBS and Alström syndrome (ALMS, MIM #203800), the genotyping microarray has facilitated the molecular diagnosis by the rapid detection of known variations in the main genes related to them [59, 60], but however limits the identification of novel mutations or novel candidate genes. These tests turned up no results. Conversely, WES enabled us to identify potential causative mutations in all families, even after applying strict criteria for data filtering as proposed by other authors [61].

The suspected clinical diagnosis of BBS was confirmed in two patients (cases 2 and 3) by identifying two nonsense mutations in *BBS2* gene. In addition, a mutation in *ALMS1*, the only gene associated with ALMS to date, was found in case 4, a patient suspected of having BBS. Since these positions are not included in the Asper Ophthalmics genotyping microarray and *BBS2* is not a predominant gene according our cohort, these mutations could not be detected in our initial screening. It is well-known that both syndromes show a significant phenotypic overlap [7, 62, 63], primarily early-onset obesity, diabetes and retinal dystrophy. This fact is consistent with the idea that both entities could share similar molecular etiologies, so it is no wonder that patients can be clinically misdiagnosed, although recent efforts are being made to clarify the mechanisms underlying these syndromes [62, 63].

After exploring the involvement of known ciliary genes, three cases remained without a clear molecular diagnosis. Although case 1 was clinically diagnosed with BBS since the patient showed five primary features commonly related to this syndrome, no mutation was found either in any *BBS* gene or in other ciliopathy-related genes. This led us to believe in the possibility of a novel candidate gene responsible for the phenotype displayed by this patient. Thus, an alternative filtering strategy revealed *CORO2B* as the main candidate causative gene in this family. *CORO2B* encodes Coronin-2B protein, which seems to play a role in the reorganization of neuronal actin structure (GeneCards database, http://www.genecards.org/, identifier GC15P068559). Although it is predominantly expressed in brain, it can also be found in other human tissues such as retina, heart, kidney, adipocytes and reproductive organs (GeneCards), which are commonly affected in many ciliopathies. In addition, this protein contains several WD-repeat domains, frequently found in intraflagellar transport proteins (IFT80, IFT122, IFT172), dynein-related proteins (DNAI1, DNAI2, DYNC1I1) and other proteins associated with ciliogenesis (POC1A, POC1B) [64, 65]. Proteins containing these WD-repeat domains are thought to play important roles in the assembly of multiprotein complexes involved in the majority of pathways in eukaryotic cells, this being the reason why this kind of proteins are commonly associated with numerous genetic diseases [66]. Interestingly, the p.(Leu194Gln) mutation found in this gene localizes in one of these WD40-repeat domains. The fact that PPIs tools have shown the co-expression and physical interaction of Coronin-2B with well-known ciliary proteins via a third protein, together with the identification of Coronin-2B in ciliary studies in other organisms, reinforces our hypothesis of *CORO2B* as the main candidate gene for this family.

A similar situation occurs with case 5, with a clinical diagnosis of MKKS. After ruling out the presence of mutations in *MKKS*, the only gene linked to the disease to date, and other ciliopathy genes, only two possible candidate genes remained: *ZNF17* and *LMO7*. To date, there is little information about these two genes. *ZNF17* encodes the zinc finger protein 17, which may be involved in transcriptional regulation (GeneCards, identifier GC19P057411) and is evolutionary conserved across species. This protein seems to be co-expressed with KIAA0753/OFIP [53], whose gene has been recently identified as causative in a patient with Oral-facial-digital VI syndrome and forms a ternary complex with OFD1 and FOR20 proteins, which are necessary for basal body anchoring and, consequently, for a non-defective primary cilia ciliogenesis. This, together with its association with centrosome and pericentriolar satellites in human cells, made us think in a possible involvement of ZNF17 in ciliogenesis. The second gene, *LMO7*, encodes the LIM-only 7 protein, which is known to be involved in PPIs(GeneCards, identifier GC13P075620). It seems to take part in the same pathway as ACTN2, which in turn physically interacts with TTC8, often related to BBS and retinitis pigmentosa (Genemania). Additionally, LMO7 seems to participate in regulation of actin cytoskeleton as part of the adherens junction pathway (KEGG Pathway database, http://www.genome.jp/kegg/pathway.html), thus the involvement of LMO7 in ciliary disease would not be unlikely. Given the above-mentioned information, and considering that both proteins are expressed in multiple human ciliated tissues such as brain, retina, heart, adipocytes and reproductive organs (GeneCards), it would not be rare to find these proteins playing an important role in ciliogenesis and/or ciliary function, but further studies will be needed to confirm their involvement in ciliary pathogenesis.

The two cases described above clearly show the genetic and phenotypic complexity of ciliary diseases, leading to misdiagnosis in some instances. Thus, case 5 seemed to be a phenotypic overlap between two syndromes. This child was clinically diagnosed with MKKS because of the symptoms displayed at the first assessment but no mutation was found in *MKKS* gene, so we decided to review our data in search of mutations in other *BBS* genes, since it is well-known that many patients diagnosed with MKKS during neonatal period or early childhood may turn out to be a case of BBS years later, developing retinal dystrophy and obesity [67, 68]. However, it was not possible to find causative mutations either in *BBS* or in other ciliopathy-related genes, which led us to seek and propose novel genes as candidates. Case 6 also turned out to be a case of misdiagnosis. While the studied patient was initially referred to as a case of BBS with retinal dystrophy and obesity, the recent reassessment of the phenotype only confirmed the presence of retinal dystrophy, which can be explained by defective *CRB1* expression, and thus indicating that the previously diagnosed obesity was nonsyndromic. By contrast, her heterozygous sibling evolved from polydactyly into a more complex phenotype, suggesting that although *CRB1* might be the causative gene in the studied patient it would be necessary to perform WES in the sibling with the more severe phenotype. This highlights the importance of a full clinical evaluation and, above all, a patient follow-up for providing an accurate molecular diagnosis, prognosis and efficient genetic counselling.

Anyway, we cannot discard the presence of pathogenic variants in known ciliopathy-related genes that are not likely to be detected in WES, such as variants in non-coding parts of the transcripts, promoters, or long distance regulatory elements. In addition, some types of variants, such as copy number variations (CNVs), structural variants (SVs), middle-sized indels and deep intronic variants may be missed [10, 69]. While some of these issues could be addressed better with whole genome sequencing (WGS), this approach entails higher costs and the large volume of data to analyse requires more advanced pipelines to filter and detect disease-causing variants, which made it less suitable for clinical application [70]. Given the consanguinity of the families included in this study, the combination of homozygosity mapping and WES would have been a good strategy to follow, since it allows us to identify loss of heterozygosity (LOH) regions where homozygous disease-causing variants are frequently located [71, 72]. Instead, we obtained ROHs from WES data and confirmed that all candidate variants are located in ROH regions. This has been described as a useful approach to identify deleterious mutations in consanguineous families, particularly those with low frequency in general population [73].

In summary, we show WES as a useful and cost-effective tool for molecular discovery and diagnosis in families with suspected ciliopathy phenotype, allowing us to identify three homozygous mutations, one of them novel, which confirm two cases of BBS and another of ALMS. In addition, the strategy we adopted has also enabled us to propose novel candidate genes in two families, which seem to be potentially related to cilia. Future functional studies will be needed to understand in depth the functional impact of all the variants identified herein and also to verify the possible role of the novel candidate genes in the molecular pathogenesis of ciliopathies, thus giving new insights into cilia biology.

## Supporting information

S1 Table List of common ciliary genes used as training set for prioritisation with Endeavour and ToppGene Suite tools. (DOCX)

S2 Table ROH regions identified from WES data. Abbreviations: ROH, runs of homozygosity.
(DOCX)
