# Supplementary material for: Whole exome sequencing as a diagnostic tool for patients with ciliopathy-like phenotypes
Source: PLoS One. 2017 Aug 11;12(8):e0183081. doi: 10.1371/journal.pone.0183081 (PMC5553726; doi:10.1371/journal.pone.0183081)
Supplement: S1 Table — (DOCX) [file pone.0183081.s001.docx]

**S1 Table.** List of common ciliary genes used as training set for prioritization with Endeavour and ToppGene Suite tools

| Gene | Transcript ID (Ensembl) | Chromosome location | Transcript Length (base pairs) | Related ciliopathies |
| --- | --- | --- | --- | --- |
| *BBS1* | ENST00000318312 | 11q13 | 3423 | Bardet-Biedl syndrome |
| *BBS2* | ENST00000245157 | 16q21 | 2963 | Bardet-Biedl syndrome |
| *ARL6* | ENST00000335979 | 3q11.2 | 1390 | Bardet-Biedl syndrome Retinitis pigmentosa |
| *BBS4* | ENST00000268057 | 15q24.1 | 2490 | Bardet-Biedl syndrome |
| *BBS5* | ENST00000295240 | 2q31.1 | 3475 | Bardet-Biedl syndrome |
| *MKKS* | ENST00000347364 | 20p12.2 | 2539 | Bardet-Biedl syndrome McKusick-Kaufman syndrome |
| *BBS7* | ENST00000264499 | 4q27 | 3752 | Bardet-Biedl syndrome |
| *TTC8* | ENST00000345383 | 14q31.3 | 2183 | Bardet-Biedl syndrome Retinitis pigmentosa |
| *PTHB1* | ENST00000242067 | 7p14.3 | 4027 | Bardet-Biedl syndrome |
| *BBS10* | ENST00000393262 | 12q21.2 | 3595 | Bardet-Biedl syndrome |
| *TRIM32* | ENST00000373983 | 9q33.1 | 3688 | Bardet-Biedl syndrome |
| *BBS12* | ENST00000314218 | 4q27 | 3244 | Bardet-Biedl syndrome |
| *MKS1* | ENST00000393119 | 17q23 | 2392 | Bardet-Biedl syndrome Joubert syndrome Meckel syndrome |
| *CEP290* | ENST00000552810 | 12q21.32 | 7948 | Bardet-Biedl syndrome Joubert syndrome Leber congenital amaurosis Meckel syndrome Senior-Loken syndrome |
| *WDPCP* | ENST00000272321 | 2p15 | 3392 | Bardet-Biedl syndrome |
| *SDCCAG8* | ENST00000366541 | 1q43 | 2567 | Bardet-Biedl syndrome Senior-Loken syndrome |
| *LZTFL1* | ENST00000296135 | 3p21.31 | 4073 | Bardet-Biedl syndrome |
| *ALMS1* | ENST00000264448 | 2p13.1 | 12922 | Alström syndrome |
| *TMEM216* | ENST00000515837 | 11q13.1 | 1955 | Meckel syndrome Joubert syndrome |
| *TMEM67* | ENST00000453321 | 8q22.1 | 4651 | Meckel syndrome Joubert syndrome Nephronophthisis |
| *RPGRIP1L* | ENST00000379925 | 16q12.2 | 5297 | Meckel syndrome Joubert syndrome |
| *CC2D2A* | ENST00000503292 | 4p15.3 | 5175 | Meckel syndrome Joubert syndrome |
| *NPHP3* | ENST00000337331 | 3q22.1 | 4362 | Meckel syndrome Nephronophthisis Senior-Loken syndrome |
| *TCTN2* | ENST00000303372 | 12q24.31 | 2910 | Meckel syndrome Joubert syndrome |
| *B9D1* | ENST00000261499 | 17p11.2 | 908 | Meckel syndrome Joubert syndrome |
| *NPHP1* | ENST00000393272 | 2q13 | 2752 | Senior-Loken syndrome Nephronophthisis with Leber Amaurosis |
| *NPHP4* | ENST00000378156 | 1p36.31 | 4994 | Senior-Loken syndrome Nephronophthisis |
| *IQCB1* | ENST00000310864 | 3q13.33 | 2594 | Senior-Loken syndrome |
| *OFD1* | ENST00000340096 | Xp22.2 | 3611 | Oral-facial-digital syndrome type 1 |
| *RP1* | ENST00000220676 | 8q12.1 | 7100 | Retinitis pigmentosa |
| *RPGR* | ENST00000318842 | Xp11.4 | 3108 | Retinitis pigmentosa |
| *PKD1* | ENST00000262304 | 16p13.3 | 14138 | Polycystic kidney disease (autosomal dominant) |
| *PKD2* | ENST00000237596 | 4q22.1 | 5056 | Polycystic kidney disease (autosomal dominant) |
| *PKHD1* | ENST00000371117 | 6p12.3-p12.2 | 16282 | Polycystic kidney disease (autosomal recessive) |
| *DNAI1* | ENST00000242317 | 9p13.3 | 2499 | Primary Ciliary Dyskinesia |
| *DNAH5* | ENST00000265104 | 5p15.2 | 15633 | Primary Ciliary Dyskinesia |
| *DNAH11* | ENST00000328843 | 7p15.3 | 14188 | Primary Ciliary Dyskinesia |
| *DNAI2* | ENST00000311014 | 17q25.1 | 2236 | Primary Ciliary Dyskinesia |
| *TXNDC3* | ENST00000199447 | 7p14.1 | 2312 | Primary Ciliary Dyskinesia |
| *RSPH9* | ENST00000372163 | 6p21.1 | 2534 | Primary Ciliary Dyskinesia |
| *RSPH4A* | ENST00000229554 | 6q22.1 | 2825 | Primary Ciliary Dyskinesia |
| *INVS* | ENST00000262457 | 9q31.1 | 3709 | Nephronophthisis |
| *GLIS2* | ENST00000433375 | 16p13.3 | 3705 | Nephronophthisis |
| *NEK8* | ENST00000268766 | 17q11.2 | 3581 | Nephronophthisis |
| *AHI1* | ENST00000265602 | 6q23.3 | 4335 | Joubert syndrome |
| *IFT80* | ENST00000326448 | 3q25.33 | 4306 | Short-rib polydactyly syndrome |
| *EVC* | ENST00000382674 | 4p16.2 | 7063 | Ellis–van Creveld syndrome |
| *EVC2* |  | 4p16,2 |  | Ellis–van Creveld syndrome |
| *GUCY2D* | ENST00000254854 | 17p13.1 | 3698 | Leber congenital amaurosis Cone-rod dystrophy |
| *RPE65* | ENST00000262340 | 1p31.3-p31.2 | 2610 | Leber congenital amaurosis Retinitis pigmentosa |
| *DYNC2H1* | ENST00000398093 | 11q22.3 | 12945 | Short-rib polydactyly syndrome |
