# Supplementary material for: Whole exome sequencing as a diagnostic tool for patients with ciliopathy-like phenotypes
Source: PLoS One. 2017 Aug 11;12(8):e0183081. doi: 10.1371/journal.pone.0183081 (PMC5553726; doi:10.1371/journal.pone.0183081)
Supplement: S2 Table — Abbreviations: ROH, runs of homozygosity. (DOCX) [file pone.0183081.s002.docx]

|  | ROH (threshold for consanguinity 100,000 kb) | Gene | Variant | Mutation inside a ROH | Homozygous region containing the variant |
| --- | --- | --- | --- | --- | --- |
| Case 1 | 129,940 | *SLC3A1* | p.(Tyr461His) | Yes | 44187829-45640334 (1,452,506 kb) |
|  |  | *CORO2B* | p.(Leu194Gln) | Yes | 68510770-72941381 (4,430,612 kb) |
| Case 2 | 289,463 | *BBS2* | p.(Arg189*) | Yes | 46491414-58757683 (12,266,270 kb) |
| Case 3 | 570,650 | *BBS2* | p.(Tyr644*) | Yes | 55862836-62859956 (6,997,121 kb) |
| Case 4 | 151,366 | *ALMS1* | p.(Arg2669*) | Yes | 46231525-86259443 (40,027,919 kb) |
| Case 5 | 223,637 | *LMO7* | p.(Pro297Leu) | Yes | 73650117-82264343 (8,614,227 kb) |
|  |  | *ZNF17* | p.(Glu635*) | Yes | 57060573-59093484 (2,032,912 kb) |
| Case 6 | 167,407 | *CRB1* | p.(Ile205Aspfs*13) | Yes | 196888799-199356219 (2,467,421 kb) |

**S2 Table. ROH regions identified from WES data**

Abbreviations: ROH, runs of homozygosity.
